# Virtual barriers: unpacking the sustainability implications of online food spaces and the Yellowknife Farmers Market’s response to COVID-19

**DOI:** 10.1186/s12937-021-00664-x

**Published:** 2021-01-29

**Authors:** Josalyn Radcliffe, Kelly Skinner, Andrew Spring, Lise Picard, France Benoit, Warren Dodd

**Affiliations:** 1 School of Public Health and Health Systems, University of Waterloo, Waterloo, Canada; 2 Laurier Centre for Sustainable Food Systems, Wilfrid Laurier University, Waterloo, Canada; 3 Yellowknife Farmers Market, Yellowknife, NWT Canada

**Keywords:** Farmers markets, Sustainability, Food system, COVID-19, Yellowknife, Northwest Territories

## Abstract

**Background:**

Through their support of local agriculture, relationships, and healthy diets, farmers markets can contribute to a sustainable food system. Markets like the Yellowknife Farmers Market (YKFM) are social spaces that support local food, yet the COVID-19 pandemic has forced changes to their current model. We explore the potential of online marketplaces to contribute to a resilient, sustainable food system through a case study of the YKFM.

**Methods:**

In 2019, a collaborative mixed-method evaluation was initiated by the YKFM and university partners in the Northwest Territories (NWT), Canada. The evaluation included an in-person Rapid Market Assessment dot survey and questionnaire of market patrons from two YKFM dates prior to the pandemic. Due to COVID-19, a vendor survey and interviews were deferred. Data collected from the two patron surveys, alongside researcher observations, available literature, public announcements, and informal email and phone discussions, inform the discussion.

**Results:**

For the patron surveys, 59 dot survey and 31 questionnaire participants were recruited. The top motivators for attendance were eating dinner, atmosphere, and supporting local businesses, and most patrons attended as couples and spent over half of their time talking to others. The YKFM did not move online; instead, they proposed and implemented a “Shop, don’t stop” market. Informal conversations suggested the small scale of the market and technology challenges were perceived barriers to moving online. The physically-distanced market was well-attended and featured in local media.

**Conclusions:**

NWT food strategies rely on farmers markets to nurture a local food system. Data suggest a potential incongruence between an online model and important market characteristics such as the event-like atmosphere. Available literature suggests online markets can support local food by facilitating purchasing and knowledge-sharing, yet they do not replicate the open-air or social experience. The decision not to move online for the YKFM reflects market patron characteristics and current food context in Yellowknife and the NWT. While online adaptation does not fit into the YKFM plan today, online markets may prove useful as a complementary strategy for future emerging stressors to enhance the resiliency of local systems.

## Background

A sustainable food system in the Canadian North requires a transformation to a self-reliant and just system that supports health through equitable and secure access to nourishing foods [1–5]. Long before COVID-19 limited travel and gatherings, the Northwest Territories (NWT) has been impacted by high levels of food insecurity due to complex issues of remoteness, development and governance [6, 7]. While climate change and extreme weather continues to have profound impacts on access to and availability of both store-bought and traditional foods, climate change is also expanding the potential for growing local food in the NWT and building a strong sustainable agricultural system that supports human health within planetary boundaries is critical to the region [8–10]. As the COVID-19 pandemic exposes gaps in the current global food system, Canada released a joint statement alongside other nations stating that food security depends on local resilience and supporting small-scale farmers, harvesters, and processers with planting, harvesting, and the fair and safe sale of products [11]. Farmers markets, like the Yellowknife Farmers Market (YKFM), have been ambitiously situated as a means to support a sustainable local food system and a mechanism to promote prosperity [12–14].

Farmers markets are defined by their capacity to build direct connections and ‘short circuit’ the conventional food system by bringing farmers and community members together in the sale of locally-produced food [3, 12, 15]. These markets are no longer, however, considered a panacea to solve all the environmental and social problems of conventional agriculture and food systems [5, 16, 17]. They are community food spaces with the potential to connect communities and producers, provide infrastructure to support small sustainable farms, and support resilient integrated systems that are essential in times of crisis [11, 12, 16–18].

**Fig. 1:**
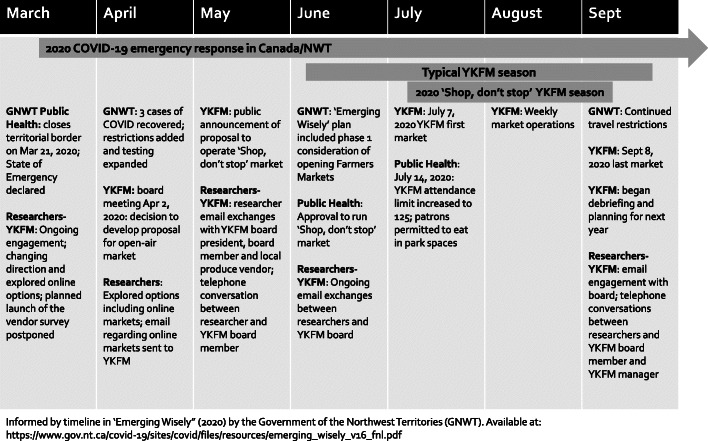
Timeline of key events related to the COVID-19 pandemic in the NWT

The COVID-19 pandemic was a significant challenge requiring local food systems to evolve and adapt. During the planning stages for the 2020 YKFM season, emergency orders began March 18, 2020 from the Government of the NWT that prohibited feasts and gatherings [19]. The resulting uncertainty led to the exploration of alternatives to the open-air, in-person YKFM model. Supported by new agriculture funding for e-commerce, many markets across Canada considered a transition to online platforms to adapt during the pandemic [20–23]. In June 2020, the Government of the NWT released their strategy titled “Emerging Wisely” which included consideration for allowing open-air, in-person farmers farkets as part of Phase 1, the first stage of relaxing COVID-19 gathering restrictions [24]. A timeline of key events related to the COVID-19 pandemic in the NWT is included as Fig. 1. The uncertainty regarding the future of the YKFM and increasing reliance on online food shopping across Canada due to national public health restrictions, prompted the authors to begin an exploration of the potential for online markets to contribute to a resilient, sustainable local food system [17].

The sustainability implications of in-person alternative food systems and markets have been heavily discussed and critiqued; however, there is much less discussion regarding online markets and alternative food spaces [25, 26]. Moreover, there is little literature that compares or explores online spaces with the lens of resiliency and sustainability. Our research joins an emerging discussion regarding the potential for these online spaces to contribute to sustainability in the face of system shocks like the COVID-19 pandemic [27, 28].

We have framed our discussion regarding online food markets and sustainability through a case study of the YKFM. The case study was informed by the most recent available patron data collected as part of a collaborative evaluation, documents posted online by the YKFM that detail their pandemic response, and informal emails and telephone conversations as a result of ongoing engagement between the YKFM and academic partners. We integrate literature with our case study to explore how online markets may contribute to building a self-reliant, sustainable local food system, and the contributing factors that led the YKFM to forego moving online.

### Online farmers markets

A small number of studies have described the potential for online markets to share knowledge regarding production and products, enable small-scale sales and connect with consumers at pick-up or delivery [25, 26, 29–31]. Wills and Arundel explored characteristics of online and offline shoppers in Australia and Canada and found that online shoppers were more motivated by cost-saving. However, shoppers in both types of markets were similarly privileged in terms of their access to time, financial, and knowledge resources [25]. Ulsperger and Ulsperger’s qualitative study of an online market in Arkansas concluded that convenience was a driving factor in online sales, and that, while producers valued connections with consumers, the depth of connection was limited at product pick-up [26]. Schumilas anchored her discussion of the need for open source technology solutions for the sustainable food movement through the open-source platform of the Open Food Network [30]. The Open Food Network hosts food markets, hubs, and individual producers online with attention to food and data sovereignty [29, 30]. Schumilas and Prost explored issues of governance and food democracy with the system and considered the Open Food Network as an opportunity to aggregate resources with local producers that did not inherently address food security or challenged the conventional food system [29, 30]. This small body of literature points to the limits of online shopping to foster connections and reproduce some of the valued characteristics of farmers markets.

While offline, in-person interactions in alternative food spaces have been found to be important for building trust, transparency, and deeper connections between consumers and farmers [25, 26, 31, 32], some studies have explored the potential for online community building [33]. Bos and Owen described the potential for ‘virtual reconnection’ through social media and online knowledge sharing that fostered trust and online connections between consumers and local food producers [32]. In a cross-sectional study comparing online and in-person knowledge-sharing in Italy, De Bernardi linked online knowledge sharing with fostering sustainable food purchasing and consumption [31]. While not speaking specifically regarding online food markets, Johnson described farmers markets as a leisure space that fostered a community of consumption and emphasized that community does not need to be geographically fixed and asserts that online communities can be as real as offline [33]. Thus, while not an inherent feature of online markets or broader online local food spaces, virtual spaces were described as having the potential to foster ‘real’ community based on caring, shared interest, and consumption ethics [29, 30, 32, 33].

### Farmers markets and the COVID-19 pandemic

There is some limited research regarding online markets and food system shocks. After hurricane Katrina in New Orleans, online markets permitted sale of urban-cultivated small crops and products to provide economic benefit to the producer and community as well as increase access to local options [18]. Research is also emerging that assesses the effect of the COVID-19 pandemic on farmers markets and food systems. Worstell evaluated market response in the context of ecological resilience and, alongside an article by Klisch and Soule, drew attention to the innovation demonstrated by markets in the United States that adapted rapidly and used the established farmers market Coalition to share information and resources [27, 28]. Online businesses and markets were found to be well-positioned to respond to the pandemic. Worstell emphasized how COVID-19 demonstrated that rapid shifts in the global food system in response to crises (such as climate change) are possible [28]. In a review of the market impacts of the pandemic, Richards and Rickard stated that increases in online purchasing may be a lasting trend [34]. The open-source Open Food Network platform (http://openfoodnetwork.ca) in Canada experienced increased uptake in response to pandemic-disrupted food distribution and access [22]. Through the COVID-19 pandemic, the need for accessible online place-based knowledge specific to the local climate and growing conditions has also increased alongside growth in home gardening and subsistence agriculture with potential to increase local access to fruits and vegetables [11, 28].

## Methods

### Case study: the Yellowknife Farmers Market (YKFM)

#### Local context

The YKFM began in 2013 as a marketplace to “promote and grow the local food system” [35]. A volunteer Board of Directors oversees the market and projects that support food-based economic development. The YKFM also supports local growers and advocates for social and political change to address food security, including the creation of a Yellowknife Food Charter [14, 35, 36]. The YKFM runs in a rented outdoor public space where vendors sell primarily baked goods, meals, fruit and vegetables, fish, birch syrup, art and housewares for 2 hours each Tuesday evening from June until September. The YKFM board has also supported a wide variety of programs including gardening advice, cooking with local ingredients, and supporting small-scale vegetable and fruit producers to participate through donating or selling their products through a collective ‘Harvester’s Table’ at the market. As part of its goals to address food security needs, the market implemented a voucher program for low income residents of Yellowknife through local social service agencies. YKFM vendors are defined as ‘local’ insofar as they live within and grow, harvest, or process their products within the NWT, although exceptions may be made if the outside vendor is meeting local needs [37]. Observations and conversations suggested that food and products are largely produced in the area although ingredients or materials were often sourced elsewhere (e.g., yarn and wood used in housewares and art; noodles and flour in prepared foods).

#### Collaborative process

The YKFM has developed close relationships to university-based researchers with past projects supported and evaluated with the assistance of academic partners at Wilfrid Laurier University [14]. In 2019, the YKFM put out a call for assistance with an evaluation that would inform the future management of the market, waste reduction efforts, and consider ways to coordinate the ‘Harvester’s Table.’ Additional goals included defining the social and economic role of the market in the community and exploring community needs and preferences. Academic partners from the University of Waterloo and Wilfrid Laurier University answered the call and supported the evaluation and re-envisioning with a plan for a utilization-focused evaluation that engages YKFM patrons, vendors, and the broader community [38]. The patron surveys discussed here reflect the first stage of this collaborative evaluation in summer 2019. During the autumn and winter that followed into 2020, the team created an online vendor survey that was planned to be released in March 2020 followed by vendor interviews. Ethics approval was obtained by both Wilfrid Laurier University and the University of Waterloo for the surveys.

#### Patron surveys

In the summer of 2019, survey questions were selected through a collaborative process between the YKFM board and university partners. The patron survey question development process was informed by a scan of published farmers market evaluation reports and best practices as well as past YKFM surveys that had been conducted by the YKFM board [39, 40]. Online markets were not a board consideration when the survey was implemented, therefore questions to assess patron response to them were not included. The resulting data collection strategy used two complementary approaches – a dot sticker survey and a paper-based questionnaire - to gather data from patrons attending the YKFM during two 2019 market dates, one in August and the other in September. Survey questions asked patrons to provide information regarding their habits and intentions for that day to reduce potential recall bias.

The evaluation team was set up at a common entry and exit point; patrons were recruited from passersby and by one researcher actively recruiting while walking through the market. Both the dot survey and paper questionnaire were offered in English and utilized convenience sampling with no remuneration beyond surveyor gratitude. The sample therefore reflects a non-random proportion of market patrons. To foster confidentiality and reduce social desirability bias, participants completed the questionnaire survey on their own. The survey included demographic questions but did not request any personal identifiers.

##### Dot survey

**Fig. 2:**
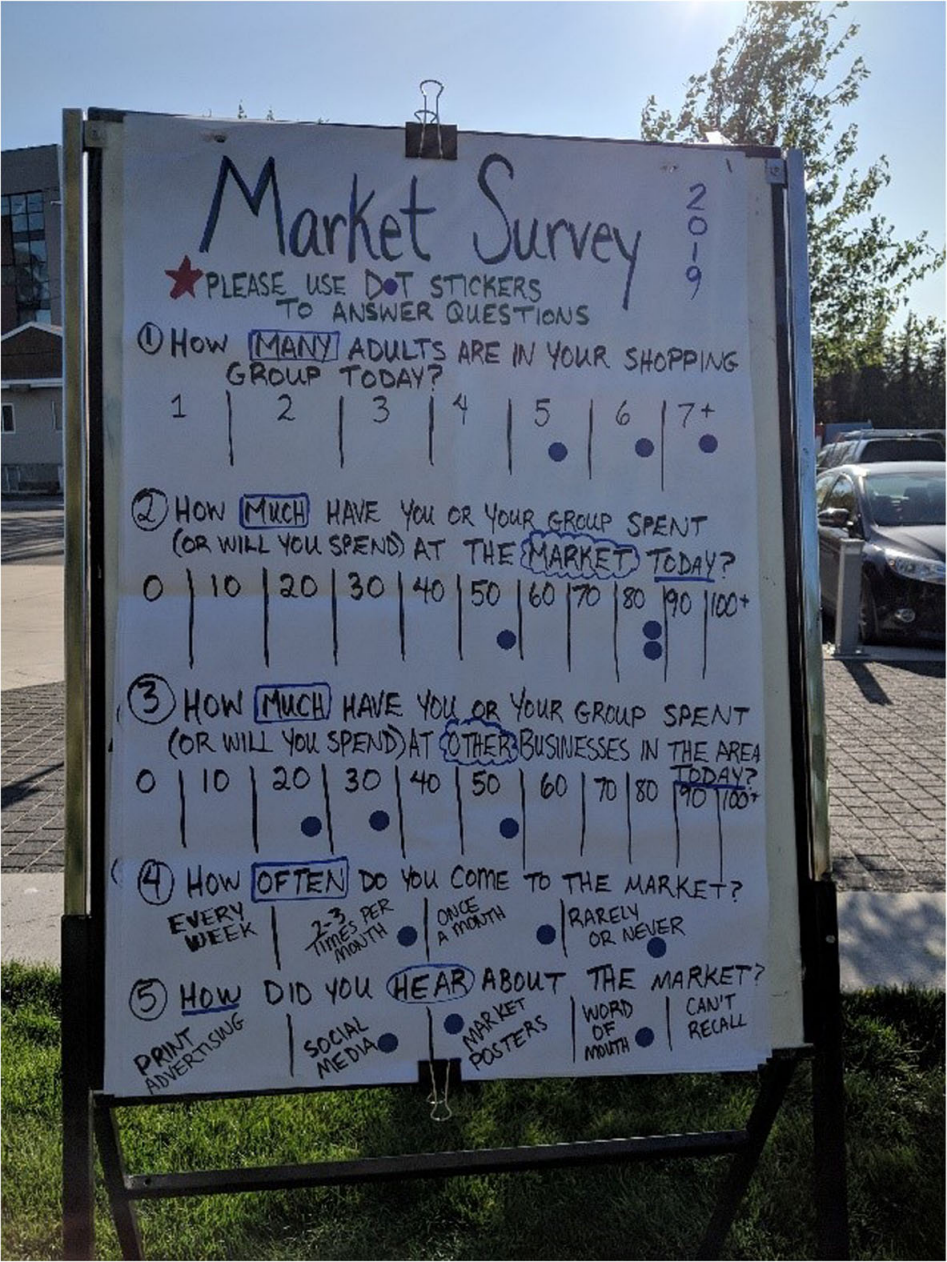
Dot survey flipchart with random ‘seeded’ responses

The dot survey was informed by a Rapid Market Assessment (RMA) methodology, which has been incorporated in market evaluations both in Canada and the United States [39, 41–43]. The dot surveys are designed to maximize the number of patrons reached with short, simple, quantitative questions with a low time burden for participants. Patrons were given five round ‘dot’ stickers and invited to place them on flipchart paper with their answers to each multiple-choice question on behalf of their group (Fig. 2). On the second market date, the chart paper was ‘seeded’ with three randomly selected dot stickers per question placed on the flipchart before patrons arrived to counter the potential social influence of seeing others’ responses [38]. Figure 2 shows the first survey chart seeded with dots that were later omitted in data analysis.

Descriptive statistics were calculated for each question from dots placed by patrons on the flipchart. Replicating a method used by a Nova Scotia Farmers Market report, average spending was calculated by first multiplying the number of dots by the spending amount in the category (e.g. 5 x $10; 13 x $20) then summing and dividing by the number of respondents [42]. As the top category used was ‘$100+,’ the averages may be an underestimation as these respondents may have spent more than $100.

##### Paper questionnaire

At the same time as the dot survey, more in-depth information was gathered from a second group of patrons who were able to contribute more time. The paper-based questionnaire included questions from past YKFM surveys and those used in published market survey reports from a national survey and the provinces of British Columbia and Nova Scotia [39, 40, 42]. Questionnaires included both demographic questions and primarily closed-ended questions, with the opportunity for open-ended feedback at the end of the survey. Descriptive statistics were then calculated for comparison to past surveys and reporting to the YKFM board. For one question (‘What two things would improve your experience at the market?’) that requested only two options be selected a weighting technique was used so that each participant who completed the question contributed an equivalent of two responses. As seen in Table 2, where participants selected less than or more than 2 options, participant contribution was weighted to equal two (i.e. if 1 was selected, this was weighted as 2; if 4 were selected, each were given a weight of 0.5).

#### COVID-19 pandemic

Throughout the months before and after the pandemic began, ongoing engagement between the university partners continued largely via email but occasionally by telephone. During spring 2019, as rising COVID-19 cases led to strict public health measures to restrict mobility, the university team based in Ontario found online markets were highlighted in the media and emerged in local food systems conversations as options to sustain local markets [20–23]. When the vendor survey was put on hold and the YKFM 2020 outdoor season became uncertain, the academic partners provided information regarding online market software that could be an opportunity for YKFM to continue to link consumers and producers for the 2020 market season. To determine the most appropriate way forward, the YKFM had conversations with vendors and board members and held virtual board meetings. These conversations took place during their typical annual vendor recruitment and onboarding period for the 2020 market season. The researchers learned informally through emails and telephone calls with a YKFM board member and the YKFM manager about concerns and considerations that arose during the process. This ongoing engagement with the YKFM informed this discussion of the YKFM response to the pandemic and the information presented was reinforced by data from online YKFM public announcements and social media posts.

## Results

### Collaborative process

With the support of the YKFM, the academic partners gathered evaluation data in-person on the survey dates in 2019. These experiences were opportunities for the academic partners to act as participant-observers and to have informal conversations with vendors, board members, and patrons that assisted later in validating the survey results. The YKFM takes place at the water’s edge facing a rocky landscape; vendors set up tents near a circular pathway surrounding a greenspace dotted with trees. Local music plays near the shore and many families bring bicycles and strollers and sit with their food on the picnic tables and greenspaces. Compost bins are strategically placed to collect leftover food and compostable food packaging from meals purchased from vendors. A diversity of languages can be heard throughout the market; Yellowknife has a rich tourism industry and is home to local Dene First Nations, French-speakers, and others bringing languages from around the Canadian North and world.

The longest lines at the YKFM were to purchase a hot meal which often sold out before the market end time of 7:15 p.m., while other non-meal vendors posted sold-out signs before the end of the market. In alignment with researcher observations, a YKFM board member confirmed that the baked goods sell out at nearly every market, while produce vendors often left with some leftovers in past years. While farmers markets in southern Canada are often characterized by the wide variety of produce available, only two tables at the YKFM (of nearly twenty) offered locally grown foods in recent years. Produce for sale included surprising offerings such as bok choy. In past years, cooking programs through the YKFM helped to ensure that community members were able to process and cook the vegetables that were available. Through both the Harvester's Table and the vendor booths, the YKFM offered a way of selling small quantities of a diverse array of foods that were not always available in conventional grocery stores.

In line with the goals of the utilization-focused evaluation, upon completion of the patron surveys, an early report of results was sent to the market for use in planning and grant applications in December 2019. Further reports that briefly highlighted key findings in the context of other surveys were produced for the market in February 2020 to inform the upcoming season. During this time, planning and refining of vendor surveys were completed with the vendor survey set for release in March 2020.

### Patron surveys

#### Dot survey

The dot survey recruitment resulted in 59 participants, answering on behalf of their groups, over the course of the two survey dates (27 participants in August, 32 participants in September). The results from the dot survey are included in Table 1. More than half of participants attended the YKFM in groups of two (55%; *n* = 32), with two-thirds attending in groups of two or more people (median group size = 2). Patrons spent similar amounts at the YKFM and surrounding businesses ($29 and $33 respectively), and approximately one fifth (*n* = 11) of the participant groups planned to spend no money at the YKFM. Word of mouth was, by far, the most selected (59%; *n* = 35) method for participants to hear of the YKFM whereas a quarter (*n* = 15) of participants chose social media.

**Table 1:** YKFM August & September 2019 patron evaluation: dot survey results^a^

**Number of adults are in your shopping group today? (*n*** **= 58)**	**Number (%)**
1	19 (33%)
2	32 (55%)
*3*	4 (7%)
*4*	1 (2%)
*5*	0
*6*	1 (2%)
*7+*	1 (2%)
**How much will you (or your group) spend (or will you spend) at the market today?** (CAD $; *n* = 57)	**Number (%)**
$0	11 (19%)
$10	5 (9%)
$20	13 (23%)
$30	12 (21%)
$40	6 (11%)
$50	2 (4%)
$60	2 (4%)
$70	1 (2%)
$80	1 (2%)
$90	0 (0%)
$100+	4 (7%)
**How much will you (or your group) spend (or will you spend) at other businesses today? (CAD $; *n*** **= 57)**	**Number (%)**
$0	10 (18%)
$10	6 (11%)
$20	8 14%)
$30	14 (25%)
$40	2 (4%)
$50	8 (14%)
$60	3 (5%)
$70	1 (2%)
$80	0 (0%)
$90	1 (2%)
$100+	4 (7%)
**How often do you attend the market? (*n*** **= 58)**	**Number (%)**
Every week	11 (19%)
2–3 times/month	19 (33%)
1 time/month	8 (14%)
Rarely/never	20 (35%)
**How did you hear about the market? (*n*** **= 59)**	**Number (%)**
Word of mouth	35 (59%)
Social media	15 (25%)
Can’t recall	4 (7%)
Print	3 (5%)
Posters	2 (3%)

^a^ As some patrons chose not to answer certain questions, different sample sizes were used for each question (57–59)

#### Paper questionnaire

The total sample of the paper questionnaire was 31 participants (11 participants on August 27 2019; 20 participants on September 10 2019). Patrons surveyed were primarily adults between 30 and 39 years of age (45%; *n* = 14) with incomes between $100,000–$140,000 CAD (35%; *n* = 11) although nearly a fifth of participants (*n* = 6) chose not to self-report their income. The majority of those at the YKFM self-identified as Yellowknife residents (81%; *n* = 25). Patron demographics are summarized in Table 2.

**Table 2:** YKFM August & September 2019 patron evaluation: paper questionnaire demographics (*n* = 31)

**Age**	**Number (%)**
18–24	0 (0%)
25–29	5 (16%)
30–39	14 (45%)
40–49	5 (16%)
50–64	5 (16%)
65+	2 (6%)
**Income ($, CAD)**	**Number (%)**
<$30,000	2 (6%)
$30,000–$49,000	3 (10%)
$50,000–$69,000	1 (3%)
$70,000–$99,000	5 (16%)
$100,000–$149,000	11 (35%)
$150,000–$199,000	2 (6%)
>$200,000	1 (3%)
Did not specify	6 (19%)
**Residency**	**Number (%)**
Yellowknife	25 (81%)
Temporary worker	2 (6%)
Tourist/visitor	4 (13%)

The self-reported motivations, behaviours, and views of patrons expressed in the paper questionnaire are presented in Table 3. Buying dinner was among the top reasons for attending the YKFM, tied with supporting local businesses and the atmosphere (58%; *n* = 18). The most common item purchased (or intended to be purchased) was baked goods (*n* = 16), while 39% (*n* = 12) reported buying or planning to buy dinner or vegetables, which aligns with earlier mentioned observations and board member comment. Local food (74%; *n* = 23) and food from the NWT (84%; *n* = 26) were important to patrons, while good weather was chosen more often by participants than nutrition (39% vs 29%; *n* = 12 vs *n* = 9). Most participants (65%; *n* = 22) reported walking or cycling to the YKFM. Socializing with friends or family and vendors made up approximately half of the time spent by participants (53%) which aligns with the dot survey finding that most patrons surveyed attend in groups of two or more (67%; *n* = 39).

**Table 3:** YKFM August & September 2019 patron evaluation: paper questionnaire patron motivations, behaviour, and views (*n* = 31)

**Reasons for attending the market**	**Number (%)** **% = # of times selected/n**
To buy and eat dinner	18 (58%)
Support local businesses	18 (58%)
Atmosphere	18 (58%)
Buy ready-to-eat meals	16 (52%)
Meet friends/socialize	15 (48%)
Buy fresh produce	14 (45%)
Support local food	14 (45%)
Spend time with friends/family	14 (45%)
Buy baked goods	12 (39%)
Buy non-food products	7 (23%)
Buy fish	7 (23%)
Meet people	7 (23%)
Hear local musicians/talent	7 (23%)
See new vendors	5 (16%)
A specific vendor	4 (13%)
A specific product	3 (10%)
To eat food from home	2 (6%)
Other^a^	2 (6%)
**Products purchased/intended to purchase**	**Number (%)** **% = # of times selected/n**
Baked goods	16 (52%)
Vegetables	12 (39%)
Food concessions^b^	12 (39%)
Specialty food^c^	8 (26%)
Dairy	6 (19%)
Pottery	6 (19%)
Preserves/spreads	5 (16%)
Fish	5 (16%)
Jewellery	4 (13%)
Fruits	2 (6%)
Birch syrup	2 (6%)
Wool/knitted products	2 (6%)
Eggs	1 (3%)
Herbs/tea/coffee	1 (3%)
Plants	1 (3%)
Soaps/creams	1 (3%)
Other arts/crafts	1 (3%)
Fresh flowers	0
Wood products	0
**Important factors for patrons when buying food at the YKFM**	**Number (%)** **% = # of times selected/n**
Grown/produced NWT	26 (84%)
Grown/produced locally	23 (74%)
Packaging/waste	15 (48%)
In season	12 (39%)
Good weather	12 (39%)
Nutrition	9 (29%)
Grown/produced Canada	9 (29%)
Price	9 (29%)
Environmental impact	8 (26%)
Fair trade	7 (23%)
Food safety	6 (19%)
Animal welfare	5 (16%)
Appearance of product	4 (13%)
Natural (not certified)	4 (13%)
Certified organic	2 (6%)
Ease of preparation	2 (6%)
Look of packaging	2 (6%)
**Improvements to improve the market experience**^**d**^	**Number (%)** **% = # of times selected/total selections (58)**^e^
More ready-to-eat foods	18 (30%)
More fresh produce	11 (19%)
Music every week	8 (13%)
More fish/meat/eggs	4 (7%)
More baked goods	4 (7%)
More beverages	4 (7%)
Indoor space for poor weather	3 (6%)
More non-food products	2 (4%)
Tables from other NWT communities	2 (3%)
More compost/waste reduction	1 (2%)
More tables from community groups	1 (1%)
More open market time	1 (1%)

^a^Other = work, music

^b^A food concession is a stall or stand where food, beverages, and edible items are sold

^c^Specialty food was not defined for participants; responses reflect their interpretation

^d^Weighted responses to equalize participant contributions

^e^Two participants did not answer this question so only 58 selections (reflecting 29 participants)

### COVID-19 response: change of plans

Amid planning for the 2020 YKFM season, COVID-19 became a rapidly growing concern which soon led to restrictions that severely limited travel to and from the NWT. Academic partners were restricted from planning and completing travel for research. While there has been widespread movement towards online spaces in Canadian provinces supported by federal funding, moving into a virtual marketplace was not the chosen approach by the YKFM [21, 22, 34, 44]. Other NWT markets in Desnedé and Hay River also implemented physically-distant markets once approved by their respective health authorities, while a number of individual market vendors in Yellowknife operated online stores [45–48]. While the NWT maintained a very small number of COVID-19 cases relative to Southern Canada, the YKFM and other local markets were delayed due to continued precautionary measures by the Government of the NWT outlined in the ‘Emerging Wisely’ plan [24].

While many non-food artisans and some local food producers had already established online stores and the board was familiar with these platforms, many reservations were discussed regarding transitioning to an online market. Researchers heard concerns from a YKFM board member related to produce vendors’ ability to meet demand from online orders given their small scale; in the 2020 season, only one produce vendor sold at the YKFM and sold out each week despite considerably increasing their offerings. The accessibility of online selling was also one of the considerations before the season began. For example, the produce vendor expressed concern regarding seniors’ access and ability to use online platforms and adapted to take orders over the phone during the 2020 season. Additionally, the risk of duplicating or competing with other local vendors that had moved online at the start of the season independently was discussed. Furthermore, the YKFM also recognized the importance of its social role, informed by the patron results, as well as its limited current ability to provide local fruits and vegetables which distinguished the YKFM from southern counterparts that were embracing online markets during the pandemic.

After deliberation, there was a decision in spring 2020 to propose an alternative market that respects physical distancing requirements that began in July alongside other similar markets across the NWT [47, 48]. Early remarks from the YKFM suggest large attendance and gratitude from patrons who attended while activities in the NWT continued to be significantly restricted. Despite the ‘Shop, don’t stop’ marketplace, the interactions within households continued in park spaces nearby. Still the ability to connect with vendors, a defining and celebrated feature of farmers markets, was most impacted as spending time paused at booths and within the YKFM area was discouraged.

## Discussion

A sustainable food system in the North serves ‘people, the planet, and profit,’ and producing local food is integral to this goal [6, 7, 10, 13, 49]. As future shocks are expected due to climate stressors, this discussion considers the research on online food spaces within the context of building a resilient, sustainable food system in the context of current place-based strengths and barriers for the YKFM. The decision of the YKFM not to pursue an online market model will be explored alongside the broader goals of the YKFM and other alternative food spaces to contribute to a socially, economically, and environmentally sustainable food system.

### Connections: social sustainability

The face-to-face interactions and tactile connection to the food are celebrated with farmers markets broadly, with literature describing patrons’ market experiences as much more than a ‘grocery trip’ but a meaningful leisure experience and opportunity to build connections [3, 26, 30, 33]. As remarked by Martin, “farmers markets are about conversations and relationships” [3] (p168). True to the reputation of farmers markets, the surveyed YKFM patrons valued local, community-grown food and products, appreciated the atmosphere, and spent their time eating dinner and talking together. The YKFM board takes care to offer an inviting space with local musicians, dinner options, picnic tables, and a view of the lake. As previously mentioned, nearly a fifth of the surveyed patrons did not come to purchase anything at all. The high priority placed on the atmosphere and eating a meal with their community sets YKFM apart from other markets across Canada where patrons attend primarily for freshness and local foods and only 2% attend for the dining options [40].

The decision of the YKFM to not pursue an online market reflects the market’s distinct features as a space to connect as a community and enjoy dinner within an event-like atmosphere, all of which would be threatened using an online model. However, the COVID-19 pandemic means that the market had to shift away from being this vibrant community space to one that supports physical distancing between participants and vendors. While the YKFM was able to preserve some face-to-face interaction within households and briefly with vendors, the physical distancing and ‘Shop, don’t stop’ motto discouraged conversations with vendors. While limited engagement continued on the YKFM’s social media page, a collective online space to explore, network and learn about products may have helped to maintain and improve the connection and engagement with the local products, vendors, and community.

At the heart of farmers markets role in a sustainable food system is their ability to foster engagement with food production and build a “civic agriculture” or deep sense of connection and social responsibility [17, 26, 29]. The transition to online markets may connect patrons to local foods and foster community around ethical consumption; however, a simple online store with individual pick-up would likely not constitute an authentic virtual community that engages food citizens [26, 29, 32]. For markets facing crises like COVID-19, however, online markets may help to efficiently connect patrons and consumers to limit physical contact and time in public spaces while informing them of the product options and sources. Even without purchasing options, markets like the YKFM can build upon existing online communities using social media or other platforms to build food skills, knowledge, connections, and resilience during system shocks, like COVID-19, that can leverage urban growing spaces and nurture self-determination and reliance [18, 29, 31, 32]. In addition, these networks can expand beyond the boundaries of Yellowknife to benefit, connect, and inform communities and food producers to support Northern self-reliance.

Online platforms do not need to exist in isolation. Between crises that limit travel such as extreme storms, wildfires or pandemics, in-person connections and communities can reinforce online spaces for a shared knowledge network that can foster adaptations that align with Northern values and food security needs such as food sharing and more equitable food distribution [6, 7, 31]. While the YKFM functions largely at a small urban scale, their ability to scale up to facilitate food access and connections with patrons and vendors in nearby rural areas may depend on building accessible virtual spaces. Remote, primarily Indigenous populations, with some of the highest food insecurity, may choose to purchase healthy local foods remotely, but this appears unlikely without an online space facilitating the ability to view and confirm products before traveling hours to pick-up [1, 7]. Thus, online markets that temporarily (or permanently) replace or complement the traditional in-person marketplace have the potential to expand access to some populations where necessary infrastructure exists. In Northern Canada, the lack of reliable rural internet and electrical systems are real barriers that contribute to the inequitable distribution of the benefits of both online communities and access to local foods in an online marketplace [6, 26, 29]. With the expectation of increased production and support for building a local food system from the Government of the NWT in the future, infrastructure investments to increase electrical and internet capacity may help address these concerns before the next system shock occurs [13].

### Building the local food system: economic sustainability

Farmers markets in the North like YKFM are critical for nurturing small food enterprise for the intertwined sustainability goals of nourishing health and self-reliance, ecologically sound approaches, and local economies. While growing and producing more food in the North is a concrete goal of local and territorial food strategies, it is also a business strategy in the North [13, 36, 50]. The concerns of YKFM regarding meeting potential online demand for vegetables and requests from patrons for more fresh produce demonstrate the current limited local production. Northern communities, including patrons of the YKFM, support and value locally-grown produce [6, 10, 51]. However, building a commercially viable food business is a challenge in the North due to limited subsidy of small operations, competition with subsidized imported foods, and limited suitable land and soil [1, 7, 51].

For the YKFM, the online model was discussed as more appropriate for larger and more stable markets such as those in southern Canada. As structural and financial supports grow in the NWT with support from the government, this may no longer be a barrier in the future. During a crisis that limits in-person interaction and disrupts transportation, online markets may sustain the connection to local food, increased access to fresh fruit and vegetables, and sustain emerging commercial producers. While an open-air summer market was deemed safe and early remarks from the YKFM suggest it was widely appreciated and supported by the community, their annual indoor Christmas market may not be approved. The YKFM can consider networking with existing online stores into a local hub for mutual benefit.

Mechanisms that sustain market organizations and food networks during crises can increase the chances that these networks will be operational to support social entrepreneurs as communities recover from shocks. Online markets and communities can also foster a more social entrepreneurship and ethical consumption by sustaining micro-farms that may otherwise not have distribution networks [13, 18]. Using existing online tools during system shocks like COVID-19, such as the YKFM website or social media accounts that are supporting home gardening, may encourage and support upcoming commercial growers and producers when in-person services are not available.

### Growing within planetary boundaries: environmental sustainability

When distribution and harvesting is disrupted due to climate change impacts and crises, online markets have the potential to sustain availability of fruits and vegetables, which is essential for improved health from the individual to planetary level [4]. Food sovereignty and local resilience depend on local food harvests and production, and environmental sustainability will depend on knowledge-sharing of growing methods that reduce land-clearing and environmental impacts [2, 4, 6, 13, 52]. The YKFM has worked to promote ecologically sustainable practices directly through the Yellowknife Food Charter, urban growing initiatives, as well as through the composting program at their events, which also produces soil needed to support local food growing [1, 10, 13, 36]. Online communities can also be considered alongside the physically-distanced marketplace of the 2020 COVID-19 pandemic to build a community around shared ethics and beliefs that can support increasing use of sustainable growing practices [6, 31, 33]. Although discussed last here, building a community and momentum towards ecologically-supportive agriculture cannot be an afterthought; it is key to achieving the dual goals of a new local food industry and sustainable development to mitigate and adapt to climate change and nurture human and animal life for generations to come [1, 2, 4, 52]. Thus, while the YKFM proposes to shift to a physically-distanced market to connect farmers and consumers, the need for community building online or offline must remain a priority.

### Limitations

These data reflect a small case study based on a collaborative evaluation. While the collection of pre-pandemic patron data in-place is a strength to mitigate recall bias, the data must be considered as a snapshot of patrons in the context of previous surveys, informal YKFM board and management email and telephone conversations, observations, reports and literature. As online markets were not considered in the patron surveys, patron attitudes regarding virtual markets could not be assessed directly. Furthermore, some survey items were non-discrete (i.e., local food and food produced in the NWT; food concessions and ‘ready-to-eat’ food) and this may have influenced responses. However, the survey represents the most recent available data regarding the YKFM to inform a place-based discussion regarding the potential for online marketplaces as adaptations to the current COVID-19 pandemic. Survey responses in the questionnaire largely aligned with findings from literature and surveys in Yellowknife and surrounding Northern regions. Future exploration is warranted to explore civic or alternative food network responses to the pandemic and future shocks as well as the role of virtual spaces in fostering resilient sustainable food systems in the NWT and beyond.

## Conclusions

Patrons at the YKFM value the community experience and local foods offered by the YKFM and building a local food system in the NWT is widely supported by the government, researchers, YKFM board, and patrons. However, this system must be adaptable and resilient as it faces today’s COVID-19 pandemic and predicted future shocks. With many converging challenges in the North driving momentum towards a more socially, economically, and environmentally sustainable food, online markets and communities can be valuable and innovative tools to sustain local production and continue to build production capacity. As a small scale market offering an event-like open-air atmosphere, the YKFM did not transition to an online marketplace in response to the 2020 pandemic; nevertheless, virtual food spaces are expanding and will be valuable tools to meet the challenges of tomorrow.

## Data Availability

The data that support the findings of this study are controlled and permitted by the Yellowknife Farmers Market. Data are available from the authors upon reasonable request and permission of the Yellowknife Farmers Market.

## Abbreviations

COVID-19: Coronavirus Disease 2019
NWT: Northwest Territories
YKFM: Yellowknife Farmers Market
